# *RET* mutation p.S891A in a Chinese family with familial medullary thyroid carcinoma and associated cutaneous amyloidosis binding *OSMR* variant p.G513D

**DOI:** 10.18632/oncotarget.4992

**Published:** 2015-08-22

**Authors:** Xiao-Ping Qi, Jian-Qiang Zhao, Zhen-Guang Chen, Jin-Lin Cao, Juan Du, Nai-Fang Liu, Feng Li, Mao Sheng, Er Fu, Jian Guo, Hong Jia, Yi-Ming Zhang, Ju-Ming Ma

**Affiliations:** ^1^ Department of Oncologic and Urology Surgery, the 117th PLA Hospital, Wenzhou Medical University, Hangzhou 310004, Zhejiang Province, China; ^2^ Department of Head and Neck Surgery, Zhejiang Cancer Hospital, Hangzhou 310022, Zhejiang Province, China; ^3^ Zhejiang Academy of Medical Sciences, Hangzhou 310007, Zhejiang Province, China; ^4^ Institute of Dermatology, Chinese Academy of Medical Sciences and Peking Union Medical College, Nanjing 210042, Jiangsu Province, China; ^5^ Department of Dermatology, the 117th PLA Hospital, Wenzhou Medical University, Hangzhou 310004, Zhejiang Province, China

**Keywords:** thyroid neoplasia, medullary thyroid carcinoma, cutaneous amyloidosis, RET mutation, OSMR variant

## Abstract

There are no reports on the relationship between familial medullary thyroid carcinoma (FMTC) associated with cutaneous amyloidosis (CA) and *RET* or *OSMR/IL31RA* gene mutations. In this study, we investigated a Chinese family with FMTC/CA and found a recurrent *RET* c.2671T>G (p.S891A) mutation in six of 17 family members. Three of the six p.S891A mutation carriers presented with medullary thyroid carcinoma (MTC). Of them, three (two with and one without MTC) were diagnosed as having combined lichen/macular biphasic CA. We also identified a novel *RET* variant, c.1573C>T (p.R525W) in five members. Of them, three carriers had no evidence of thyroid/skin or basal serum/stimulated calcitonin abnormalities. *In vitro* cell proliferation assay indicated that oncogenic activity of *RET* p.S891A was slightly enhanced by p.R525W, whereas p.R525W alone had no effect on cell proliferation. Meanwhile, we identified a novel *OSMR* variant, c.1538G>A (p.G513D) in seven members. We noticed that three *OSMR* p.G513D carriers presenting with CA also had the *RET* p.S891A mutation. Our investigation indicated that the *RET* p.S891A mutation combined with *OSMR* p.G513D may underlie a novel phenotype manifesting as FMTC and CA.

## INTRODUCTION

Medullary thyroid carcinoma (MTC) occurs as a sporadic condition in approximately 75% or in an inherited form as a component of the type 2 multiple endocrine neoplasia (MEN 2) syndromes, MEN 2A and MEN 2B, and familial MTC (FMTC) in 25% of all cases [1]. As one subtype of MEN 2, FMTC is operationally diagnosed in families with four or more cases of MTC in the absence of pheochromocytoma or hyperparathyroidism, which occurs in 10–20% of all MEN 2 cases with an older age at onset (often between 20 and 40 years) [1]. FMTC is regularly correlated with germline mutations of the *RET* proto-oncogene, which is mapped on chromosome 10q11.2 and contains 21 exons [2]. The *RET* gene encodes a tyrosine kinase transmembrane receptor characterized by three different domains: the extracellular domain, the transmembrane domain, and the intracellular tyrosine kinase domain [3, 4]. Current data indicate that a confirmable mutation has been described in almost all MEN 2 families [1]. Germline mutations in FMTC kindreds are more equally distributed across the *RET* gene and include mutations at codons 532, 533 (exon 8); 609, 611, 618, 620 (exon 10); 630, 634 (exon 11); 768, 790, 791 (exon 13); V804M, 844 (exon 14); 891 (exon 15); and 912 (exon 16). Mutations at codons 532, 533, 768, 844, and 912 have been identified only in families with FMTC [1]. Recently, some compound mutations of *RET* (p.V804M/V778I, p.V804M/R844L, and p.C634Y/Y791F) have been reported to have some specific clinical characteristics [1, 5–14]. Additionally, some rare cases have also been reported to be associated with specific *RET* mutations in many MEN 2 families, such as cutaneous lichen amyloidosis (CLA), Hirschsprung's disease (HSCR), and corneal nerve thickening (CNT) [1, 5, 6, 11, 15–17]. In 2009, the American Thyroid Association (ATA) stratified mutations at codon 891 as ATA-A level, carrying the “least high” risk [1].

The CLA phenotype in MEN 2 usually appears on the upper back as a subtype of cutaneous amyloidosis (CA), and most of the previous cases of CLA in MEN 2A have been anecdotally described [1, 18–29]. The clinical presentation of this MTC-CLA was initially observed only in MEN 2A patients with mutations in the extracellular cysteine 634 codons in exon 11. Another exception is the p.V804M mutation within exon 14, which is in an intracellular tyrosine kinase domain reported in a US female with MTC/CLA [27]. Familial CA mainly includes three clinical types: CLA, macular amyloidosis, and nodular amyloidosis. The pathogenic gene for familial CA is mapped to a locus on 5p13.1-q11.2. Subsequently, *OSMR*, within this locus, was demonstrated to be the causative gene for familial CA. The *OSMR* gene encodes oncostatin M receptor *OSMRβ*, which is an interleukin (IL)-6 family cytokine receptor [30–34].

In this study, we investigated a southeastern Chinese family with FMTC and CA and screened the entire coding sequence of *RET* in the available family members. We also analyzed the *in vitro* oncogenic potential of the two *RET* variants and sequenced the *OSMR* and *IL31RA* genes in this family. Finally, we evaluated the correlation between the genotype and phenotype and its potential clinical significance.

## RESULTS

### Clinical features and phenotypic data

#### Patients with MTC

**Figure 1:**
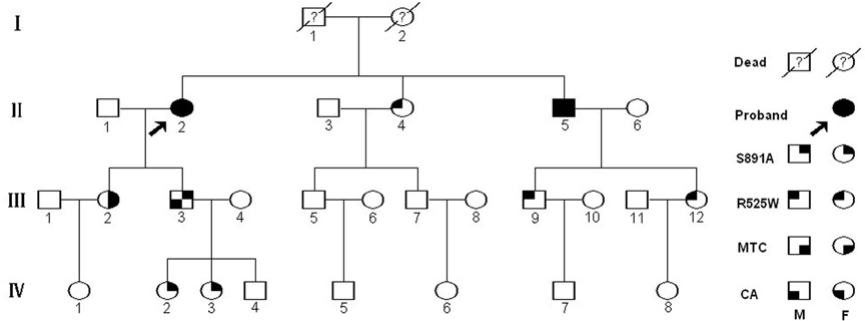
Pedigree of the southern four-generation Chinese family with FMTC and lichen/macular biphasic cutaneous amyloidosis investigated in this study Circles and squares denote female and male family members, respectively.

The proband (II-2, Figure 1) was a 65-year-old woman with diarrhea for 10 years who was then diagnosed as having MTC in 2012. Biochemical examination revealed an increased level of carcinoembryonic antigen (CEA) (29.7 ng/mL; normal, <5 ng/mL). Doppler ultrasound (USS) and computerized tomography (CT) scanning indicated two hypoechoic nodules in both thyroid lobes with right lymph node enlargement. A bilateral total thyroidectomy (TT) with bilateral level VI and right neck dissection was performed. The histopathologic evaluation suggested bilateral MTC with right neck lymph node metastases (LNMs) (T1N1bM0; Table 1). Two months later, the 45-year-old daughter of the proband (III-2) also underwent TT with bilateral level VI and modified left neck dissection after diagnosis of bilateral thyroid masses with left lymph node enlargement and an elevated CEA level (22.8 ng/ml). Bilateral MTC with LNMs was confirmed by histopathologic examination (T1N1bM0). In 2013, some previously hesitant members of the family (II-4, II-5, III-3, III-5, III-7, III-9, III-12, IV-1~7, and IV-8) agreed to further participate in biochemical testing, imaging studies, and *RET* screening (Table 1). All of the newly recruited subjects had normal basal serum calcitonin (bCt) levels and USS/CT images, with the exception of II-5 (p.S891A/p.R525W; bCt, 589.6 ng/L [normal for males, < 8.4 ng/L; normal for females, <5.0 ng/L]; CEA, 41.53 ng/ml). Then, the 58-year-old brother of the proband (II-5) accepted and underwent a TT with bilateral level VI and modified bilateral neck dissection. The histopathologic examination revealed bilateral multifocal MTC with LNMs (T1N1bM0; Table 1).

**Table 1:** Clinical characteristics of 9 *RET* mutation/variant carriers

Patient No.	Gender/Age	*RET* Mutation/variant	Pre-/Post-Ct (ng/L)	sCt(ng/L)	Surgery	Histology (MTC, L/R, cm)	LN+/resected^a^	pTNM	CA	*OSMR* Variant
II-2 (proband)	F/66	p.S891A/p.R525W	NA/<2.0	NA	TT +BiLND(VI) +MBiND	0.2/0.9	5/81	T_1_N_1b_M_0_	Yes	p.G513D
II-4	F/64	p.R525W	2.3	25.9	−	−	−.	−	−	p.G513D
II-5	M/58	p.S891A/p.R525W	589.6/3.53	NA	TT +BiLND(VI) +MBiND	1.1/1.0	14/85	T_1_N_1b_M_0_	Yes	p.G513D
III-2	F/46	p.S891A	NA/5.7	NA	TT +BiLND(VI) +MLND	1.4/0.3	5/28	T_1_N_1b_M_0_	−	p.G513D
III-3	M/44	p.S891A	3.31	62.4	WA	−	−	−	Yes	p.G513D
III-9	M/32	p.R525W	<2.0	2.8	−	−	−	−	−	−
III-12	F/30	p.R525W	<2.0	<2.0	−	−	−	−	−	p.G513D
IV-1	F/23	−	−	−	−	−	−	−	−	−
IV-2	F/21	p.S891A	<2.0	25.1	WA	−	−.	−	−	−
IV-3	F/7	p.S891A	2.16	30.2	WA	−	−	−	−	−
IV-4	M/5	−	−	−	−	−	−	−	−	p.G513D

F, female; M, male; pre-Ct, pre-operative calcitonin (normal male <8.4 ng/L and female <5.0 ng/L); post-Ct: post-operative calcitonin; sCt, calcium-stimulated Ct (normal <100 ng/L); NA, not available; TT, total thyroidectomy; MR(L)ND, modified right (left) neck dissection; MBiND, modified bilateral neck dissection; BiLND(VI), bilateral level VI lymph node dissection; WA, watchful awaiting; MTC, medullary thyroid carcinoma; pTNM, tumor stage; CA, cutaneous amyloidosis, -:negative or not available;

a LN+ includes positive lymph nodes proven on histopathology; resected includes lymph node resected.

Six other carriers harboring a *RET* p.S891A mutation or p.R525W variant (III-3, IV-2, and IV-3 or II-4, III-9, and III-12, respectively) were further submitted to stimulated calcitonin (sCt) testing. The peak value (normal, <100 ng/L) was obtained 2 min after calcium stimulation. The mean sCt was 36.40 ng/L (range, 21.7–62.4 ng/L) in the 3 *RET* p.S891A mutation carriers (mean age, 24 y; range, 7–44 y) and < 2.0, 2.8, or 25.9 ng/L in the 3 *RET* variant p.R525W carriers (mean age, 42 y; range, 30–64 y; Table 1). None of the 6 carriers had abnormal Ct values.

#### Patients with CA

**Figure 2:**
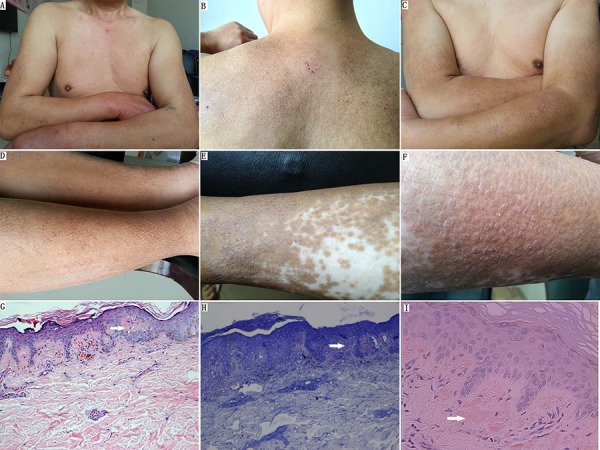
Clinical and histological presentation of FMTC/CA **A–D.** Macular amyloidosis: brown hyperpigmented macules on the upper back, shoulders, arms, and legs. (A) Postoperative neck scar and brown hyperpigmented macular skin lesion on the arms (individual II-5). (B–D) Macular amyloidosis: macules showing pigmentation with a rippled or reticulated pattern and with scabby scratches on the upper back and extremities (individual III-3). **E.** Lichen amyloidosis: dry, scaly, and thickened SKIN with hyperpigmentation and remnant white patches on the lower leg (individual II-5). **F.** Close-up of lichen amyloidosis: multiple monomorphic skin-colored and clustered lichenified papules with white thin scales on the lower leg (individual II-5). **G.** Small hyaline deposits of amyloid were situated in the papillary dermis. There is overlying epidermal hyperplasia (hematoxylin & eosin; original magnification, × 100). **H.** Globular deposits of amyloid were positive for crystal violet staining (original magnification, × 200). **I.** The papillary dermal deposits were positive for Congo red stain (original magnification, × 400).

Patients II-2, II-5 (p.S891A/p.R525W), and III-3 (p.S891A) were diagnosed as having CA by a dermatologist. None of the other 14 relatives had pruritus or skin lesions. In these three patients, the first symptoms of CA typically began on the lower legs as severe pruritus. The ages at diagnosis were 28, 27, and 31 years, respectively (Figure 1). Once pruritus appeared, the areas with lesions were repetitively scratched and subsequently developed into local skin lichenification and hyperpigmented brown papules. The papules spread to other sites of involvement in the lower legs to thighs, the upper back, shoulders, arms, and forearms (Figure 2A–2F). All three patients also presented with brown macular lesions with a rippled or reticulated appearance on the extremities and the upper back, suggesting the co-existence of both lichen and macular variants (Figure 2A–2D). Additionally, patient II-5 manifested clearer skin lesions with dry, scaly, thickened, and clustered papules, and white patches, which caused epidermal cell damage due to repeated scratching on both lower legs (Figure 2E, 2F). All three patients were treated with glucocorticoid cream, which resulted in a decreased period of itching, but the application was discontinued due to side effects. The histopathologic examination of the skin lesions showed that the overlapping epidermis was hyperkeratotic with mild acanthosis and elongation of rete ridges. Characteristic small globular deposits of amorphous eosinophilic acellular material were present in the papillary dermis (Figure 2G). Little chronic inflammatory infiltrate was noted in the dermis. Globular deposits of amyloid were positive with crystal violet and Congo red staining (Figure 2H, 2I).

#### Other MEN2-associated disease

The 17 individuals had no evidence of pheochromocytoma, hyperparathyroidism, CNT, HSCR, or other endocrine tumors.

### Identification of the *RET* germline mutations/variants

**Figure 3:**
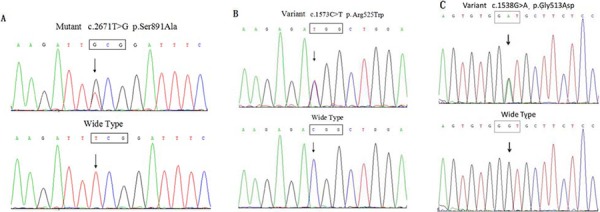
*RET* and *OSMR* variants in this pedigree were confirmed by direct sequencing **A.** Direct sequencing of PCR products from the proband demonstrated a heterozygous T-to-G substitution at nucleotide position 2671 within exon 15, resulting in a missense mutation designated p.S891A. **B.** *RET* p.R525W (c.1573C > T) within exon 8. **C.** *OSMR* p.G513D (c.1538G > A) within exon 11 was confirmed.

A heterozygous missense mutation within exon 15 of *RET*, c.2671T>G (p.S891A), was confirmed in the proband (II-2) and five other members (II-5, III-2, III-3, IV-2, and IV-3; Figure 3A). Another *RET* novel variant, p.R525W (c.1573C>T), within exon 8 was identified in five individuals (II-2, II-4, II-5, III-9, and III-12). Two patients with MTC (II-2 and II-5; Figure 3B and Table 1) underwent *RET* compound mutations (p.S891A/p.R525W). Individuals III-9 and III-12 only carry p.R525W, and this compound mutation did not co-segregate with MTC, so we can conclude that p.S891A/p.R525W passed in a *trans* inherited pattern. Meanwhile, we found five recurrent exonic single nucleotide polymorphisms (SNPs): c.135A > G (p.A45A), c.1296A > G (p.A432A), c.2071G > A (p.G691S), c.2307T > G (p.L769L), and c.2712C > G (p.S904S) within exons 2, 7, 11, 13, and 15, respectively. A SNP in intron 2 (IVS2+9A > G) was also identified (Figure 1; data not shown).

Additional screening indicated that two *RET* p.S891A mutation carriers with CA (II-5 and III-3; Figure 3C) also carry heterozygous *OSMR* c.1538G > A (p.G513D/exon10). Several *OSMR* SNPs, i.e., p.D535N (c.1657G > A/exon11), p.N703N (c.2019C > T/exon13), and p.T732T (c.2196G > A/exon14), and *IL31RA* SNPs p.P24P (c.72C > T/exon 2) and p.S529N (c.1586G > A/exon13) were also identified and are listed in Supplementary Table 1. Further sequencing of exon 10 of *OSMR* identified p.G513D in four of the other eight affected individuals with *RET* mutations/variants and one unaffected member (IV-4; Table 1).

### Functional significance of *RET* mutants (p.S891A/p.R525W and p.R525W)

**Figure 4:**
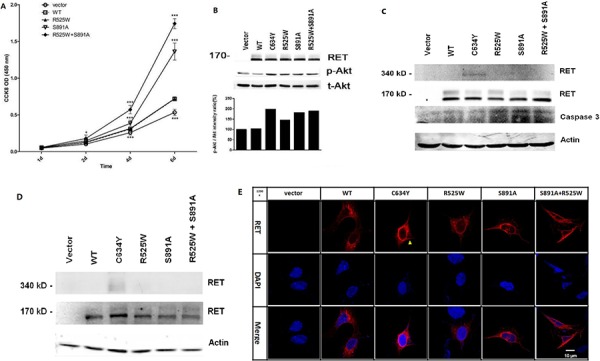
*In vitro* assays performed to characterize *RET* mutants p.R525W, p.C634Y, and p.S891A/p.R525W **A.** The promotion effect of RET and its mutants on HEK293 proliferation. *P < 0.05; ***P < 0.001. **B.** Upper panel: Western blotting was performed in HEK293T cells expressing RET and mutants using antibodies against RET, phospho-Akt (Ser473), and Akt. Lower panel: Relative phosphorylation levels were calculated as ratios of phosphorylated to total protein levels using densitometry. **C.** Western blot shows complete inhibition of glycosylation in wild-type (WT), p.R525W, p.S891A, p.S891A/p.R525W, and p.C634Y. β-actin was used as a loading control. **D.** Presence of active disulfide-bound RET homodimers in p.R525W, p.S891A, p.S891A/p.R525W, and p.C634Y cells in comparison to *RET-*WT cells. **E.** Immunostaining showed that *RET* p.C634Y (red) were mainly localized in the cell membrane and cytoplasm (yellow arrow), whereas p.R525W, p.S891A, and p.S891A/p.R525W were mainly located in the cytoplasm and rarely in the membranes. Original magnification, × 1200; scale bar, 10 um.

The transforming capacity was assessed in HEK293 and HEK293T cells transfected with wild type-*RET*, p.R525W, and p.S891A/p.R525W. We found that *RET* p.S891A/p.R525W and p.S891A had a significant effect on the promotion of cell proliferation rates, and p.S891A/p.R525W showed a stronger effect than p.S891A (Figure 4A). *RET* p.R525W, p.S891A, p.S891A/p.R525W, and p.C634Y all increased the level of Akt phosphorylation. The phosphorylation level of p.R525W was 20%, 23%, and 30% weaker than that of p.S891A, p.S891A/p.R525W, and p.C634Y, respectively (Figure 4B). To confirm whether the proliferative effect resulted from apoptosis, we detected any alteration in an important apoptosis marker, caspase 3. Western blotting indicated that caspase 3 was also downregulated by RET and its mutants (Figure 4C). RET glycosylation was completely inhibited by p.R525W, p.S891A, p.S891A/p.R525W, and p.C634Y (Figure 4C). Disulfide-bridge-mediated RET dimerization was observed only in p.C634Y mutants (Figure 4D, 4E), which is consistent with the previous conclusion that *RET* mutations affecting extracellular cysteines lead to constitutive dimerization [35], and the p.S891A and p.S891A/p.R525W mutants together functioned as a monomeric receptor. Immunostaining indicated that *RET* p.R525W, p.S891A, and p.S891A/p.R525W were located mainly in the cytoplasm but rarely in the cellular membranes, suggesting an effect on the location and thus the function of RET by these mutations. Collectively, these data indicated that *RET* p.S891A can facilitate cell proliferation through promotion of the anti-apoptotic effect of Akt and incoming mitogenic stimulators. Meanwhile, the oncogenic activities of *RET* p.S891A are lower than those of p.C634Y.

## DISCUSSION

In this study, we explored a recurrent intracellular p.S891A mutation and a novel extracellular variant p.R525W of *RET* in a southern Chinese family with FMTC/CA. All three patients with CA had the *RET* p.S891A mutation and a novel *OSMR* variant p.G513D, which provide possible new insight into the mechanism underlying FMTC/CA.

Previous studies have shown that the p.S891A mutation accounts for < 5% of all patients with *RET* mutations. Most patients with the p.S891A mutation have manifested FMTC, whereas just a few have manifested as MEN 2A [11]. Recently, 93 carriers of the p.S891A mutation were summarized [11, 12, 36]: MTC was present in 74.2% (69/93), and pheochromocytoma, hyperparathyroidism, and CNT were present in 3.2% (3/93). The mean age at MTC diagnosis in those carriers was 42.1 years. Age-related penetrance of MTC in 36 patients with the p.S891A mutation was 20.0%, 71.4%, 92.3%, and 100.0% in those aged 0–20, 21–40, 41–60, and > 60 years, respectively [11]. In our study, three of the six p.S891A mutation carriers presented with MTC as the sole clinical endocrine tumor, whereas the other 3 carriers (mean age, 24 y; range, 7–44 y) still had consistently undetectable elevations in bCt or sCt and chose a watchful waiting approach to treatment [12, 36, 37]. The clinical data in this family is consistent with that in previous patients with the *RET* p.S891A mutation and other FMTC patients with ATA-A level mutations carrying “moderate risk” (ATA-MOD) as reported worldwide recently [1, 37].

Two patients (II-2 and II-5) with *RET* p.S891A/p.R525W presented with MTC, whereas three carriers with p.R525W (II-4, III-9, and III-12) had no evidence of thyroid/skin or bCt/sCt abnormalities (Figure 1 and Table 1). There are mutations in the extracellular cysteine domain of RET that are reported to cause comparatively mild FMTC or FMTC/MEN 2A, such as p.C515S, p.C531R, and p.G533C [6, 9, 10, 14]. In this study, it is still inclusive that *RET* p.R525W is causative for MTC. *RET* double mutations associated with MEN 2 were also previously reported to have specific clinical characteristics [14]. For example, it appears that p.V778I, p.Y806C, and the p.Y791F polymorphisms have additive effects to p.V804M and p.C634Y, whereas p.R844L has an inhibitory modifying effect on p.V804M [14]. Although the oncogenic activity of p.S891A was slightly enhanced by p.R525W, two patients with *trans* p.S891A/p.R525W in our study only presented with MTC, which shows similar clinical features to p.S891A described previously [11, 12, 36]. Nonetheless, the follow-up study needs to be validated to avoid misinterpretation and irreversible clinical outcomes [38].

CLA is verified to be rarely associated with the MEN 2-related specific *RET* genotype. Eng *et al*. reported that the frequency of CLA in MEN 2A families was approximately 9% (18/199), and all 18 families were frequently associated with *RET* codon 634 mutation, but there was no specific analysis of the correlation between the *RET* genotype and CLA phenotype or of case distribution of CLA in each family [15]. In 2009, a p.V804M *RET* germline mutation was identified in a MTC/CLA subject [27]. Accumulating evidence indicates that CLA occurred in 42.3% (33/78) of 78 *RET* mutation carriers in 14 MEN 2/CLA families, similar to the approximately 50% morbidity for pheochromocytoma in MEN 2A [1, 15, 39]. Gender-related predominance in the prevalence of CLA was observed as indicated by the male-to-female ratio of approximately 2:9 (6:27) (Table 2). The mean age at the time of diagnosis of CLA with a *RET* mutation was 31.4 y (range, 5–60 y). Pruritus seems to be the first clinical manifestation of CLA because of its early onset in infancy or adolescence, and most patients present with pruritic symptoms before the diagnosis of MTC [23, 26, 40].

**Table 2:** Clinical data of patients with an association between the specific *RET* mutations and CLA in MEN 2

*RET* Mutation	Families *(NO.)*	MEN2 Subtype	*RET* carriers *(NO.)*	CLA *(NO.)*	ADC (yr)	Skin lesion of CLA	Gender (M/F)	MTC/PHEO				^a^References
								MTC *(NO.)*	ADM *(yr)*	PHEO *(NO.)*	ADP *(yr)*	
p.C634Y	1	MEN2A	10	4	NA	Interscapular region	0:4	3	NA	4	NA	^b^Ceccherini *et al* 1994 [17]
p.C634R	1	MEN2A	14	4	NA	Interscapular region	1:3	NA	NA	NA	NA	^b^ Hofstra et al 1996 [19]
	1	MEN2A	8	2	47/18	Interscapular region	0:2	2	23/12	2	23/NA	^b^Hofstra *et al* 1996 [19]
p.C634G	1	MEN2A	2	2	NA	Back	0:2	2	54/44	1	45	Seri *et al* 1997 [20]
p.C634Y	1	MEN2A	2	1	24	Upper back	1:0	1	25	1	35	Karga *et al* 1998 [40]
p.C634W	1	MEN2A	5	4	60/46/28/27	NA	1:3	4	NA	2	NA	Lemos *et al* 2002 [21]
p.C634R	1	MEN2A	1	1	21	Upper back	0:1	1	21	1	21	Vieira *et al* 2002 [22]
p.C634R	2	MEN2A	14	5	15 to 56	Interscapular, scopular region, central region of the thorax	1:4	5	NA	1	NA	^b^Verga *et al* 2003 [23]
p.C634Y	1	MEN2A	12	4	5 to 52	Interscapular, scopular region, central region of the thorax	2:2	3	NA	2	NA	^b^ Verga et al 2003 [23]
p.C634Y	1	MEN2A	1	1	NA	Interscapular region	0:1	1	34	1	34	Gullu *et al* 2005 [26]
^c^p.C634	1	MEN2A/^d^ FMTC	3	3	39/14/NA	Interscapular region	0:3	3	39/14/NA	0	−	Abdullah *et al* 2004 [25]
p.C634R	1	MEN2A	3	1	24	*Upper back and shoulders	0:1	1	34	1	35	Birla S *et al* 2014 [28]
p.V804M	1	^d^FMTC	3	1	50	Interscapular region	0:1	1	51	–	–	Rothberg *et al* 2009 [27]
**Total**	**14**	**MEN2A/FMTC**	**78**	**33**	Mean: 31.4*	**Interscapular region and/or shoulders**	**6:27**	27*	Mean: 31.9*	16*	Mean: 32.2*	
p.S891A	1	FMTC	6	3	27/28/31	^†^Upper back and legs, arms, shoulders	2:1	3	65/58/45	–	–	This study

CLA, cutaneous lichen amyloidosis; ADC, age at dignosis of CLA; F, female; M, male; MTC, medullary thyroid carcinoma; PHEO, pheochromocytoma; ADM, age at diagnosis of MTC; ADP, age at diagnosis of PHEO.

a English literatures limited to CLA and *RET* mutations;

b Detailed clinical data shown in REF 17;

c Only qualitative but not genotype;

d Suspicious case;

* available data.

* hypopigmented macular lesions;

† cutaneous biphasic amyloidosis with lichen and macular cutaneous amyloidosis.

In the present family, of the three patients with CA, the two with the p.S891A/p.R525W mutation (II-2, and II-5) had MTC, whereas the p.S891A carrier (III-3; 44 years old) did not but did have a high Ct level (Table 1). Moreover, another patient with the p.S891A mutation with MTC (III-2; 46 years old) and five carriers (three individuals with p.R525W and two individuals with p.S891A) had no CA lesions and skin pruritus. Family members with the same *RET* mutation may have different clinical phenotypes, and younger individuals might show the CA phenotype earlier [23, 40]. Our observations also indicated that the driving course of CA was independent from the clinical evolution of the disease and was not associated with MTC [23, 26, 27]. The phenotype of CA co-segregated with *RET* p.S891A implied that the germline *RET* p.S891A mutation possibly caused the FMTC/CA. SNPs within non-hot spot regions show no association with MTC or CA, which indicated that genetic screening of *RET* hot spot regions is adequate for the diagnosis [1, 6, 15]. Similar to CNT, CA appears to be another rare clinical characteristic of the *RET* p.S891A mutation [11, 14, 36]. It should be noted that families with CLA only did not have the *RET* mutation [15, 19], whereas all of the patients with FMTC/CA and the *RET* p.S891A mutation have *OSMR* p.G513D. The other four *OSMR* p.G513D carriers including a 64-year-old female (II-4), had no evidence of CA (Table 1). Therefore, *OSMR* p.G513D may play a role in modifying the evolutionary process of CA with *RET* mutation. Interestingly, however, all three of these CA patients presented a more expanded pathologic region than previously described, particularly the scapular region of the upper back corresponding to dermatomes T2-T6 in MEN 2-CLA, manifesting as co-existence of papular and macular forms known as cutaneous biphasic amyloidosis (Figure 2 and Table 2) [17, 23, 30]. This could be indirect evidence that the cases presented here are more like a “neurodermatitis” or a common clinical variant of familial CA disease and that the treatment of CA is most disappointing [32, 33]. Although the underlying molecular mechanism of CA/MEN 2 pathogenesis remains largely unknown, it may occur due to the accumulation of genetic disruptions, either through errors in chromosomal replication, or the interaction of other modifying factors and different expression patterns of the same *RET* mutations, and/or through PI3K/Akt pathways to modify disease susceptibility and the clinical phenotype [26, 32, 40, 41].

## MATERIALS AND METHODS

### Subjects

We investigated a four-generation southeastern Chinese pedigree including 17 individuals with FMTC/CA from Zhejiang Province, China (Figure 1). The present study was conducted in accordance with the Helsinki Declaration and approved by the Ethics Committee of the 117th PLA Hospital (Hangzhou, China). Written informed consent was provided by all of the subjects in this study.

All individuals underwent clinical and biochemical examinations according to the published criteria in three scenarios. (i) The biochemical evaluation consisted of CEA, parathyroid hormone, and serum and/or 24-h urinary determination of catecholamines, along with USS, CT, and/or emission CT scans. Surgical thyroidectomy was performed after confirmation of a *RET* mutation and elevated bCt for the diagnosis. (ii) Levels of bCt and sCt were measured using a fully-automated chemiluminescence immunoassay (Immulite 2000 Immunoassay System; Siemens Ltd., USA). Eight carriers of the *RET* mutation/variation participated in calcium-sCt testing as follows: Ca gluconate was administered intravenously at a dose of 25 mg/kg at 10 ml/min (2.3 mg of elemental Ca), and the blood samples were obtained before and at 2, 5, and 15 min from the end of the Ca infusion via an indwelling intravenous cannula. (iii) CA was diagnosed clinically based on persistent pruritic, cutaneous papules with some scales or macular pigmentation showing a rippled or reticulated pattern and was characterized histopathologically by amyloid deposits in the papillary dermis [17, 23]. Follow-up was then carried out.

### Histopathologic analysis

The diagnosis of MTC was further confirmed by histopathology. Tumor staging was performed according to the American Joint Committee on Cancer (AJCC, 7th edition) tumor-node-metastasis (TNM) classification system [42].

The skin biopsy specimens were obtained from the cutaneous lesions of patients II-5 and III-3, embedded in paraffin wax, and fixed in formalin. Hematoxylin-eosin, crystal violet, and Congo red stains were applied to 4-μm-thick sections.

### *RET* mutation analysis

Genomic DNA was isolated from peripheral blood leukocytes of available family members as previously described (8), followed by polymerase chain reaction (PCR) amplification and sequencing of the entire exons and the flanking splice junctions of *RET, OSMR*, and *IL31RA.* One hundred unrelated healthy matched controls and 6 affected individuals with the *RET* p.S891A mutation along with matched controls were included as previously reported [12].

### Construction of expression vector

The full-length open reading frame of *RET9* was cloned to the pCIG vector. For the *RET* p.C634Y, p.R252W, p.S891A, and p.S891A/R525W mutations, we use the KOD-Plus-Mutagenesis Kit (Toyobo, Japan) following the manufacturer's protocol. The sequence and orientation of the *RET* open reading frame and its mutants were confirmed by direct sequencing.

### Cell cultures and transfections

The human embryonic kidney cell lines HEK293T and HEK293 were purchased from the American Type Culture Collection (Manassas, VA, USA). Cells were cultured in Dulbecco's Modified Eagle's Medium (Invitrogen, Grand Island, NY, USA) supplemented with 10% fetal bovine serum. Various constructs were introduced into HEK293 cells through retroviral or lentiviral infection using standard protocols. To obtain stable transfectants, the transfected cells were grown in medium with G418 (400 μg/ml), and resistant clones were confirmed using Western blotting.

### Cell proliferation assay

Cell viability was determined with the Cell Counting Kit-8 (Dojindo Molecular Technologies, Inc., Japan) following the manufacturer's protocol. Briefly, HEK293 cell were seeded into 96-well plates (2000 cells/well). Then, 10 μl of the CCK-8 solution was added to each well of each of the plates, and the plates were incubated for 2 h in an incubator at every time point (24 h, 48 h, 72 h and 96 h, respectively) The absorbance at 450 nm was measured using a microplate reader. Each experiment was independently repeated in triplicate wells three times. Statistical analysis was carried out with one-way ANOVA (GraphPad Prism 5; GraphPad Software, Inc., La Jolla, CA, USA). Results are presented as mean ± SD. *P* < 0.05 was considered as statistically significant.

### Western blotting

Cell lysates were prepared by incubating cell pellets in lysis buffer (50 mmol/l Tris-HCl, pH 8.0; 150 mmol/l NaCl, 0.5% NP-40) for 30 min on ice, followed by centrifugation at 14,000 × *g* for 15 min at 4°C. For Western blot analysis, membranes were incubated with primary antibodies for 1 h at room temperature or overnight at 4°C, followed by incubation with secondary antibodies at room temperature for 1 h. Immunoreactive bands were detected using Western blot luminol reagent (GE Healthcare, Waukesha, WI, USA). Antibodies used were anti-Caspase3 (#9664), anti-RET (#3223), anti-p-Akt (#4060), anti-Akt (#9272) (all, Cell Signaling, Boston, MA, USA), and anti-Actin (#sc-69879; Santa Cruz, Dallas, TX, USA).

### Indirect immunofluorescence

Cells grown on coverslips were stained by indirect immunofluorescence as published elsewhere [43]. Briefly, cells were incubated with primary antibody against RET and *RET* mutants (p.C634Y, p.R525W, p.S891A, and p.S891A/R525W) and then incubated with Alexa Fluor 594- (Invitrogen Molecular Probes, Carlsbad, CA, USA) secondary antibody against mouse or rabbit IgG. Cells were then counterstained with DAPI and imaged with a laser scanning confocal microscope (Fluoview FV1000, Olympus Co., Japan).

## SUPPLEMENTARY TABLE


