# Supplementary material for: RET mutation p.S891A in a Chinese family with familial medullary thyroid carcinoma and associated cutaneous amyloidosis binding OSMR variant p.G513D
Source: Oncotarget. 2015 Aug 22;6(32):33993–4003. doi: 10.18632/oncotarget.4992 (PMC4741820; doi:10.18632/oncotarget.4992)
Supplement: Supplementary file 1 [file oncotarget-06-33993-s001.pdf]

SUPPLEMENTARY TABLE

Supplementary Table S1: *RET* mutation and SNPs in members of FMTC with CA family

Individuals No.	Gender/ CA	*RET* SNPs						*OSMR*/*IL31RA* SNPs				
		p.A45A	p.A432A	p.G691S	p.L769L	p.S904S	IVS2 +9A>G	p.D535N	p.N703N	p.T732T	p.P24P	p.S529N
II-2 (p.S891A/ p.R525W)	F/CA				++	++	++	/	/	/	/	/
II-4 (p.R525W)	F/-	++	+	+	+	+		/	/	/	/	/
II-5(p. S891A/ p.R525W)	M/CA		++		+				+		++	++
III-2(p. S891A)	F/-						++	/	/	/	/	/
III-3 (p.S891A)	M/CA	++	++		++			+		+	+	+
III-9 (p.R525W)	M/–		++		+			/	/	/	/	
III-12 (p.R525W)	F/–		++		+			/	/	/	/	/
IV-1	F/–		++				++	/	/	/	/	/
IV-2 (p.S891A)	F/–		++					/	/	/	/	/
IV-3(p. S891A)	F/–		+					/	/	/	/	/
IV-4	M/–	+	+					/	/	/	/	/

M, male; F, female; +;heterogeneity; ++,homogeneity; "–"and blank, negative; "/", no testing.
